# Morphological, anatomical and physiological leaf traits of *Q. ilex*, *P. latifolia*, *P. lentiscus*, and *M. communis* and their response to Mediterranean climate stress factors

**DOI:** 10.1186/1999-3110-54-35

**Published:** 2013-09-17

**Authors:** Loretta Gratani, Rosangela Catoni, Laura Varone

**Affiliations:** Department of Environmental Biology, Sapienza University of Rome, P.le A. Moro, 5 00185 Rome, Italy

**Keywords:** Air temperature, Drought, Leaf respiration, LMA, *Myrtus communis*, *Phillyrea latifolia*, Photosynthesis, *Pistacia lentiscus*, *Quercus ilex*

## Abstract

**Background:**

Limitations to plant growth imposed by the Mediterranean climate are mainly due to carbon balance in response to stress factors. In particular, water stress associated to high air temperature and irradiance in summer causes a marked decrease in CO_2_ assimilation. Air temperature sensitivity of photosynthesis (*P*_N_) differs from that of leaf respiration (*R*_D_). *P*_N_ often decreases sharply at temperature above its optimum while *R*_D_ increases exponentially over short term rises in temperature. Nevertheless, the impact of water deficit on *R*_D_ is still far from clear with reports in literature including decreases, maintenance or increases in its rates. The ratio *R*_D_/*P*_N_ can be considered a simple approach to leaf carbon balance because it indicates the percentage of photosynthates that is respired.

**Results:**

The results underline different morphological, anatomical and physiological traits of the evergreen species co-occurring in the Mediterranean maquis which are indicative of their adaptive capability to Mediterranean stress factors. The ratio *R*_D_/*P*_N_ varies from 0.15 ± 0.04 in autumn, 0.24 ± 0.05 in spring through 0.29 ± 0.15 in winter to 0.46 ± 0.11 in summer. The lower *R*_D_/*P*_N_ in autumn and spring underlines the highest *P*_N_ rates during the favorable periods when resources are not limited and leaves take in roughly three to five times more CO_2_ than they lose by respiration. On the contrary, the highest *R*_D_/*P*_N_ ratio in summer underlines the lowest sensitivity of respiration to drought. Among the considered species, *Quercus ilex* and *Pistacia lentiscus* have the largest tolerance to low winter temperatures while *Phillyrea latifolia* and *Myrtus communis* to drought, and *Phillyrea latifolia* the highest recovery capability after the first rainfall following drought.

**Conclusions:**

The Mediterranean evergreen specie shows a different tolerance to Mediterranean climate stress factors. The predicted global warming might differently affect carbon balance of the considered species, with a possible change in Mediterranean shrublands composition in the long-term. Understanding the carbon balance of plants in water limited environments is crucial in order to make informed land management decisions. Moreover, our results underline the importance of including seasonal variations of photosynthesis and respiration in carbon balance models.

**Electronic supplementary material:**

The online version of this article (doi:10.1186/1999-3110-54-35) contains supplementary material, which is available to authorized users.

## Background

The Mediterranean Basin has long been recognized as a model region for studying global change effects on terrestrial ecosystems (Lavorel et al. 1998). Climatic models indicate that rainfall patterns are changing in the Mediterranean Basin as a consequence of the climate change, with a marked decrease up to 15-20% occurring mainly during summer, associated to an increase in the mean maximum air temperature of about 5.1°C by the end of the 21^st^ century (IPCC 2007). These changes will result in extended periods of soil moisture deficit (Hlavinka et al. 2009). Limitations to plant growth imposed by the Mediterranean climate are mainly due to plant carbon balance in response to stress factors (Galmés et al. 2007). In particular, water stress associated to high air temperatures and an excess of light during summer, may result in a chronic photo-inhibition or down-regulation of photosynthesis causing a marked decrease in CO_2_ assimilation (Zhou et al. 2010). Carbon balance depends on the ratio between photosynthesis and respiration (Lambers et al. 1998), and both these factors change in response to climatic conditions (Baldocchi and Amthor 2001). Nevertheless, they do not necessarily respond identically to changes in these conditions (De Boeck et al. 2007). Air temperature sensitivity of photosynthesis differs from that of respiration (Morison and Morecroft 2006; Way and Sage 2008; Shen et al. 2009). Photosynthesis often decreases sharply at temperatures above its optimum (Sage and Kubien 2007; Hüve et al. 2011), with most temperate species exhibiting a broad temperature optimum in the range of 15–30°C (Atwell et al. 1999; Larcher 2004), while leaf respiration increases exponentially over short term rises in temperature (Rodríguez-Calcerrada et al. 2011). In particular, the temperature sensitivity of leaf respiration is quantified using Q_10_ i.e. the proportional increase in respiration for every 10°C rise in temperature (Armstrong et al. 2006). Photosynthesis provides soluble sugar as substrates for leaf respiration (Atkin et al. 2007) and availability of respiratory substrates determines the effect of temperature on respiratory enzymes and consequently on respiration temperature sensitivity (Atkin et al. 2002; Rodríguez-Calcerrada et al. 2011). Respiration decrease depends partially on the photosynthesis decrease in response to water deficit (Gimeno et al. 2010). Under water stress a lower photosynthetic activity limits the soluble sugar availability (Pinheiro and Chaves 2011). A lower soluble sugar level may reduce the temperature sensitivity of respiration and then to cause a respiration decrease (Rodríguez-Calcerrada et al. 2011). Nevertheless, the impact of water deficits on leaf respiration is still far from clear, with reports in literature including decreases, maintenance, or increases in the rates of this process (Gimeno et al. 2010).

Since the magnitude of photosynthetic and respiratory acclimation varies among species, these processes are still poorly understood, especially under field conditions (Shen et al. 2009). In dry-land forests of the Mediterranean region, the rates of carbon loss by plant respiration often equal or exceed the rate of carbon uptake by photosynthesis during the year, except in spring and autumn, when air temperatures and water availability are favorable (Zaragoza-Castells et al. 2008; Gratani et al. 2008).

Mediterranean plant species are distributed along different gradients of water availability, according to their capacity to withstand drought (Medrano et al. 2009). Nevertheless, if dry season lasts too long, plant water deficit may negatively affect plant species capacity for carbon assimilation, as a result of the lowest photosynthetic rates and leaf surface area produced (Pereira et al. 2007). Carbon assimilation is also related to stomatal conductance with a strong impact on plant water use efficiency (i.e. the amount of water used per carbon gain) that links plant performance with water availability (Craven et al. 2013). The strength and direction of the relationship between water use efficiency and plant performance can illustrate interspecific differences in drought tolerance strategies (Craven et al. 2013).

Considering global change, variations in water supply will induce important changes in Mediterranean plant species that suffer of water scarcity, especially during drought (Llusiá et al. 2011). Different species can respond to global change by developing different mechanisms both at physiological and morphological levels. Nevertheless if the length or strength of the dry season increases, the distribution area of the species could shrink (Díaz-Barradas et al. 2010) and affect the composition of vegetation in the long-term (Gebrekirstos et al. 2011). Assessment of vegetation level vulnerability and climate change resilience require understanding of the diversity among plant species in the current vegetation, and of their growth strategies in response to fluctuating water availability (Dawson et al. 2009). A rapid adaptation to an increased aridity will be crucial for the future of many species in the Mediterranean region (Sánchez-Gómez et al. 2011). To predict how climate change might affect future Mediterranean species presence and distribution and, as a consequence, community structure and ecosystem functioning, it is essential to have a broad knowledge of which climatic factors are constraining plant species physiological traits, and how these constraints are manifested temporally (Llorens et al. 2003). Morphological adaptations as small, thick, layered leaves with high stomatal density of small size in many Mediterranean evergreen species could favor carbon gain profits over transpiration losses (Rotondi et al. 2003; Gratani and Varone 2004, 2006). *Quercus ilex* L., *Phillyrea latifolia* L., *Pistacia lentiscus* L., and *Myrtus communis* L. are evergreen shrub species largely distributed in the vegetation of the Mediterranean Basin. *Q. ilex* extends longitudinally from Portugal to Syria and latitudinally from Morocco to France (Valladares et al. 2000); it occurs in the Mediterranean maquis and forests, growing in different soil conditions and over a broad range of elevations, from the sea level to 1100 m a.s.l. (Khatouri 1992; Terradas and Savé 1992; Gratani et al. 2003). *P. latifolia* is a drought- and salt-stress-tolerant evergreen shrub species growing in the Mediterranean maquis, forests (Gratani and Bombelli 2000; Ogaya and Peñuelas 2003) and on seashore dunes, where excess soil salinity and salt spray are additional stress agents (Ogaya and Peñuelas 2003). *P. lentiscus* occurs in a wide variety of habitats, from open communities in garigue to closed ones in more mesic sites (Correia and Diaz Barradas 2000). *M. communis* is the only species of the *Myrtaceae* in the actual flora of the Mediterranean Basin (González-Varo 2010). It grows on fertile soils of warm habitats in the Mediterranean region (González-Varo et al. 2009) and in the maquis (Pignatti 1982).

The main objective of this research was to investigate morphological, anatomical and physiological leaf traits of *Q. ilex, P. latifolia, P. lentiscus* and *M. communis* and their involvement in carbon acquisition. Moreover, the ratio respiration to photosynthesis which is indicative of the capacity of plants to produce new biomass for growing and reproductive structures (Galmés et al. 2007; Millar et al. 2011) was analyzed over the year. Improving knowledge on carbon acquisition capability of the Mediterranean species will allow us to hypothesize their presence into the distribution area over the long-term, also in consideration of global change.

## Methods

### Study site and plant material

Experiments were carried out in the period from December 2009 to October 2010, on *Q. ilex*, *P. latifolia*, *P. lentiscus*, and *M. communis* shrubs (5 shrubs per species) growing in the open, under the same environmental conditions, at the Botanical Garden of Rome (41°53′53′′N, 12°28′46′′E; 53 m a.s.l.). The selected shrubs had comparable size (height = 1.36 ± 0.19 m, mean value of the considered shrubs). During the study period the selected shrubs were not watered and they received only natural rain.

### Climate

The climate of the study area was of the Mediterranean type: the mean minimum air temperature (*T*_min_) of the coldest months (January and February) was 5.3 ± 0.2°C, the mean maximum air temperature (*T*_max_) of the hottest months (July and August) was 30.9 ± 0.2°C, and the yearly mean air temperature (*T*_m_) was 16.8 ± 6.5°C. Dry period was from the beginning of June to the end of August (65.5 mm total rainfall of the period). Total annual rainfall was 708 mm, most of it occurring in autumn and in winter (Data from UCEA for the years 1995 to 2010). During the study period *T*_min_ of the coldest month (January) was 3.8 ± 3.1°C, *T*_max_ of the hottest month (July) 34.0 ± 2.2°C, and total rainfall was 709 mm, most of it occurring in winter.

### Anatomical leaf traits

Leaf thickness (L, μm) was measured by leaf sections from fresh, fully expanded sun leaves (20 per species), collected at the end of September 2010 from the selected shrubs, and measured by light microscope. Stomatal density (SD, stomata mm^-2^) was measured from nail varnish impressions (n = 20 per species) of the inferior lamina, according to Sack et al. (2003), each of them 0.5 × 1.0 cm, obtained by a Zeiss Axiocam MRc 5 digital camera (Carl Zeiss), with Axiovision AC software (Release 4.5). Stomatal pore length (SPL, μm) and width (SPW, μm) were measured on the same recorded digital images. Dimension of the stomata was used to calculate the equivalent area of the ellipsoid representing the stomatal pore area (SPA) by the following formula: (π × length × width)/4, according to Minnocci et al. (1995) and Bartolini et al. (1997).

### Morphological leaf traits

Measurements of leaf morphological traits were carried out on fully expanded sun leaves (n = 20 per species), collected at the end of September 2010. The following parameters were measured: projected fresh leaf surface area (LA, cm^2^) (excluding petioles), obtained by the Image Analysis System (Delta-T Devices, UK), and leaf dry mass (DM, mg), determined drying leaves at 80°C to constant mass. Leaf mass per unit leaf area (LMA, mg cm^-2^) was calculated by the ratio of DM and LA (Reich et al. 1992).

Leaf tissue density (LTD, mg cm^-3^) was calculated by the ratio of LMA and leaf thickness (Wright and Westoby 2002).

### Gas exchange measurements

Measurements of gas exchange were carried out using an infrared gas analyser (ADC LCA4, UK), equipped with a leaf chamber (PLC, Parkinson Leaf Chamber). Measurements were made on fully expanded sun leaves (10 leaves per species per each sampling occasion) during the study period.

Net photosynthetic rate [*P*_N_, μmol (CO_2_) m^-2^ s^-1^], photosynthetically active radiation [PAR, μmol (photons) m^-2^ s^-1^], stomatal conductance [*g*_s_, mol (H_2_O) m^-2^ s^-1^], leaf temperature (*T*_l_, °C), and leaf chamber air temperature (*T*_ch_, °C) were measured.

The *P*_N_*g*_s_, and *E* rates shown were the mean of the maximum rates for the four days measurement per month, carried out in comparable weather conditions. During gas exchange measurements, the leaf to air vapour pressure deficit (VPD_leaf_, kPa) was calculated according to Grantz (1990) as: VPD = es – ea, where es was saturated vapour pressure at leaf temperature and ea the air vapour pressure. The intrinsic water use efficiency [IWUE, μmol (CO_2_) mol (H_2_O) ^-1^] was calculated as *P*_N_/*g*_s_ ratio, according to Medrano et al. (2009).

Measurements were carried out under natural conditions, on cloud – free days (PAR ≥ 1200 μmol m^-2^ s^-1^), in the morning, from 8.00 a.m. to 12.00 p.m., according to Reich et al. (1995).

On each sampling occasion, leaf respiration rate [*R*_D_, μmol (CO_2_) m^-2^ s^-1^] measurements were carried out contemporary to *P*_N_ ones (on the same leaves) by darkening the leaf chamber with a black paper, according to Cai et al. (2005), for 30 min prior to each measurement to avoid transient post-illumination bursts of CO_2_ releasing (Atkin et al. 1998a, 1998b). The *R*_D_ rates shown were the mean of the maximum rates for the four days measured per month, carried out in comparable weather conditions. The ratio between *R*_D_ and *P*_N_ was also calculated. Q_10_ was calculated according to Rodríguez-Calcerrada et al. (2012) as: Q_10_ = **e**^10*k*^ where *k* is the slope of the linear regression between *T*_ch_ and the natural logarithm of *R*_D_ (Atkin et al. 2005).

### Statistics

Differences in the considered variables were determined by the analysis of variance (ANOVA), and Tukey test for multiple comparisons, performed using a statistical software package (*Statistica*, *Statsoft*, USA). The regression analysis was carried out to evaluate correlations among the considered variables. The principal component analysis (PCA) was carried out in order to summarise the considered anatomical (L, SD, SPL, SPA), morphological (LMA, LTD) and physiological (*P*_N_, *R*_D_, *g*_s_, *E*, IWUE) leaf traits into major components which explained their variation in the considered species.

## Results

### Anatomical and morphological leaf traits

Anatomical leaf traits of the considered species are shown in Table 1. L ranged from 311 ± 10 μm (*M. communis*) to 419 ± 25 μm (*P. latifolia*). SD varied significantly (p < 0.05): *M. communis* had the highest SD (508 ± 82 stomata mm^-2^), followed by *Q. ilex* and *P. lentiscus* (419 ± 18 stomata mm^-2^, mean value), then by *P. latifolia* (238 ± 21 stomata mm^-2^). *P. latifolia* had the highest SPL and SPA (19.5 ± 2.6 μm, 170.3 ± 31.3 μm^2^, respectively), and *Q. ilex* the lowest ones (9.1 ± 1.9 μm, 42.1 ± 12.4 μm^2^, respectively). LMA varied from 9 ± 1 mg cm^-2^ (*M. communis*) to 20 ± 1 mg cm^-2^ (*P. latifolia*) (Table 2). *Q. ilex* had the highest LTD (613 ± 40 mg cm^-3^) and *M. communis* the lowest one (304 ± 51 mg cm^-3^).

**Table 1:** **Anatomical leaf traits at full leaf expansion of the considered species**

Species	L (μm)	SPL (μm)	SPW (μm)	SPA (μm^2^)	SD (stomata mm^-2^)
*P. lentiscus*	378 ± 54 a	11.7 ± 1.3 a	7.8 ± 1.3 a	71.8 ± 12.5 a	406 ± 20 a
*P. latifolia*	419 ± 25 a	19.5 ± 2.6 b	11.1 ± 1.3 b	170.3 ± 31.3 b	238 ± 21 b
*M. communis*	311 ± 10 b	10.5 ± 2.0 ac	5.4 ± 1.0 c	45.5 ± 15.2 c	508 ± 82 c
*Q. ilex*	314 ± 59 b	9.1 ± 1.9 c	5.9 ± 0.6 c	42.1 ± 12.4 c	432 ± 34 a

L, leaf thickness; SPL, stomatal pore length; SPW, stomatal pore width; SPA, stomatal pore area; SD, stomatal density. Mean values (±SE) are shown (n = 20). Mean values with the same letters are not significantly different (Tukey Test, p ≥ 0.05).

**Table 2:** **Morphological leaf traits at full leaf expansion of the considered species**

Species	DM (mg)	LA (cm^2^)	LMA (mg cm^-2^)	LTD (mg cm^-3^)
*P. lentiscus*	229.7 ± 53.4 a	11.7 ± 2.3 a	19 ± 2 a	521 ± 57 a
*P. latifolia*	105.4 ± 11.3 b	5.2 ± 0.4 b	20 ± 1 a	472 ± 24 a
*M. communis*	31.5 ± 7.8 c	3.5 ± 0.4 c	9 ± 1 b	304 ± 51 b
*Q. ilex*	195.2 ± 35.4 d	10.1 ± 1.6 d	19 ± 2 a	613 ± 40 c

DM , leaf dry mass; LA, projected leaf surface area; LMA, leaf mass per unit leaf area; LTD, leaf tissue density. Mean values (±SE) are shown (n = 20). Mean values with the same letters are not significantly different (Tukey Test, p ≥ 0.05).

### Seasonal gas exchange and leaf respiration variations

**Figure 1:**
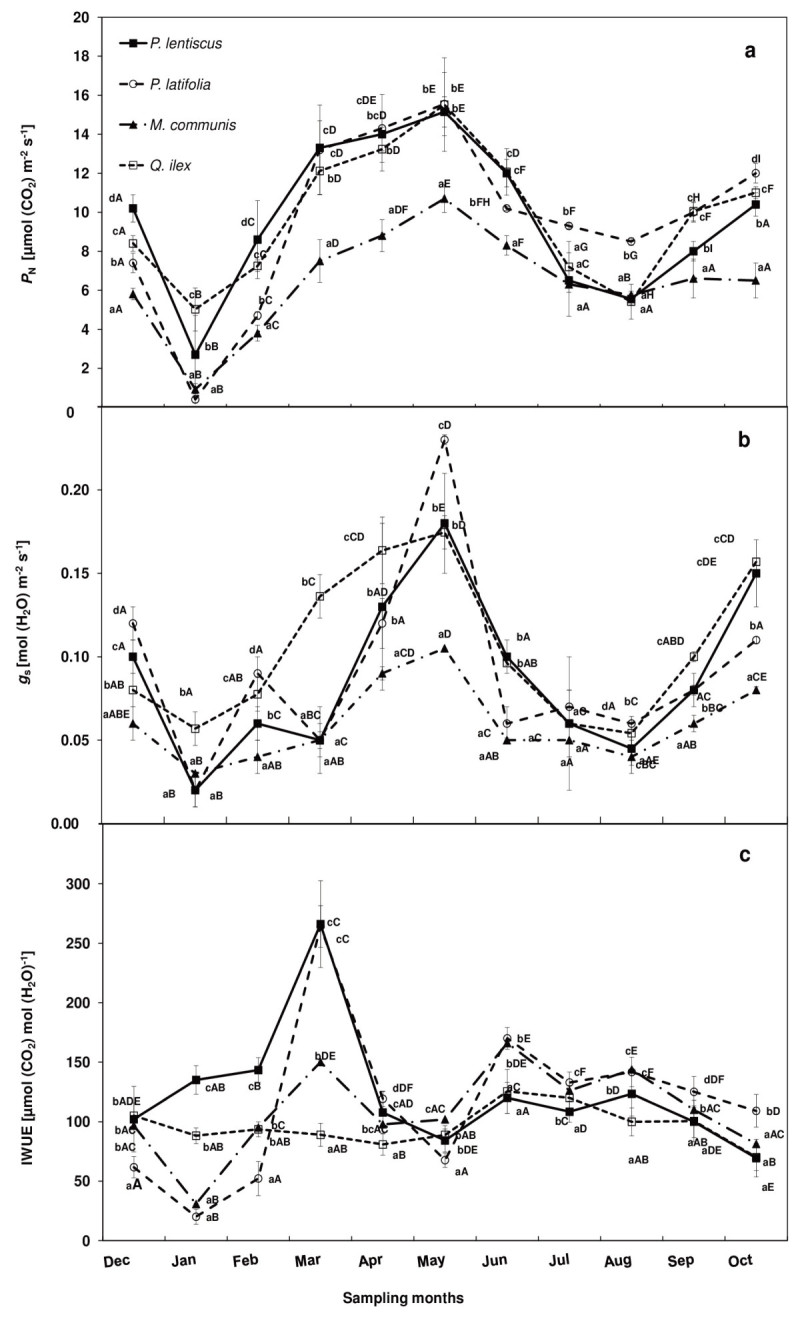
**Trend of a) net photosynthetic rate (** ***P***_**N**_**), b) stomatal conductance (** ***g***_**s**_**), c) intrinsic water use efficiency (IWUE) of** ***P. lentiscus*** **(** ***close squares*** **),** ***P. latifolia*** **(** ***open circles*** **),** ***M. communis*** **(** ***close triangles*** **), and** ***Q. ilex*** **(** ***open squares*** **) during the study period.** The mean values for each month (±SE) are shown (n = 40 leaves). Mean values with the same letters are not significantly different (p ≥ 0.05). Lowercase letters indicate the differences among the species for each month, capital letters indicate the intra-specific differences during the study period.

**Figure 2:**
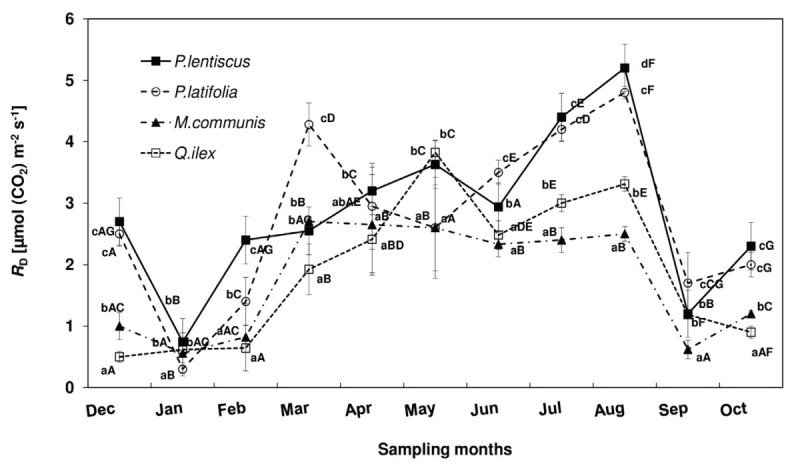
**Leaf respiration (** ***R***_**D**_**) trend of** ***P. lentiscus*** **(** ***close squares*** **),** ***P. latifolia*** **(** ***open circles*** **),** ***M. communis*** **(** ***close triangles*** **) and** ***Q. ilex*** **(** ***open squares*** **) during the study period.** The mean values for each month (± SE) are shown (n = 40 leaves). Mean values with the same letters are not significantly different (p ≥ 0.05). Lowercase letters indicate the differences among the species for each month, capital letters indicate the intra-specific differences during the study period.

Gas exchange and leaf respiration data of the considered species during the study period are shown in Figures 1 and 2.

### Spring measurements

During the study period all the considered species had the highest *P*_N_ and *g*_s_ in spring (March, April and May), peaking in May, when *T*_max_ was 23.8 ± 3.0°C, and water availability 113.8 mm (total rainfall of May). *Q. ilex*, *P. lentiscus* and *P. latifolia* had the significantly (p < 0.05) highest *P*_N_ [15.4 ± 0.2 μmol (CO_2_) m^-2^ s^-1^, mean value measured in May] than *M. communis* [10.7 ± 0.7 μmol (CO_2_) m^-2^ s^-1^].

*g*_s_ of *M. communis* [0.11 ± 0.04 mol (H_2_O) m^-2^ s^-1^, in May] was 52%, 39%, and 35% lower than *P. latifolia*, *P. lentiscus* and *Q. ilex*, respectively. *P. lentiscus* and *P. latifolia* had the lowest IWUE in May [84 ± 10 and 68 ± 6 μmol (CO_2_) mol (H_2_O)^-1^, respectively] while *Q. ilex* and *M. communis* in April [81 ± 9 and 98 ± 7 μmol (CO_2_) mol (H_2_O)^-1^, respectively].

A different *R*_D_ trend was observed in spring: *P. lentiscus* and *Q. ilex R*_D_ peaked in May [3.7 ± 0.3 μmol (CO_2_) m^-2^ s^-1^, mean value], while *P. latifolia* in March [*R*_D_ = 4.3 ± 0.6 μmol (CO_2_) m^-2^ s^-1^]. *M. communis R*_D_ was not significantly different from March to May [2.7 ± 0.1 μmol (CO_2_) m^-2^ s^-1^, mean value of the three months]. *M. communis* had the highest *R*_D_/*P*_N_ ratio (0.30 ± 0.06, mean of March, April and May), followed by *P. latifolia* (0.23 ± 0.08), *P. lentiscus* (0.22 ± 0.03) and *Q. ilex* (0.20 ± 0.05).

### Winter measurements

During winter (December, January and February) *P*_N_, *g*_s_ and *R*_D_ decreased in respect to the spring maximum, reaching the lowest rates in January (*T*_min_ 3.8 ± 3.1°C; 118.2 mm = total rainfall of the month). In particular, *Q. ilex* had the lowest *P*_N_ decrease (68% compared to the spring maximum) followed by *P. lentiscus* (82%), *M. communis* (92%) and *P. latifolia* (97%). In the same month, *Q. ilex* had the significantly (p < 0.05) highest *g*_s_ [0.06 ± 0.01 mol (H_2_O) m^-2^ s^-1^], followed by *M. communis* [0.03 ± 0.01 mol (H_2_O) m^-2^ s^-1^], *P. lentiscus*, and *P. latifolia* [0.02 ± 0.01 mol (H_2_O) m^-2^ s^-1^, mean value].

Among the species the highest IWUE was measured in *P. lentiscus* [127 ± 22 μmol (CO_2_) mol (H_2_O)^-1^, mean of December, January and February] and the lowest one in *P. latifolia* [45 ± 22 μmol (CO_2_) mol (H_2_O)^-1^]. *R*_D_ in January was, on an average, 84% lower than the spring maximum. *M. communis* and *P. latifolia* had the highest *R*_D_/*P*_N_ ratio (0.61 ± 0.02 and 0.75 ± 0.03, respectively) and *Q. ilex* and *P. lentiscus* had the lowest one (0.12 ± 0.02 and 0.27 ± 0.01, respectively).

### Summer measurements

In summer (June, July, August), *P*_N_ significantly decreased, reaching the lowest rates in August, when *T*_max_ was 32.3 ± 2.0°C, and the total rainfall of the month 4.4 mm. In particular, *P*_N_ decreased, on an average, by 64% in *P. lentiscus* and *Q. ilex*, and 46% in *P. latifolia* and *M. communis*. IWUE was lower in *P. lentiscus* (24%) and *P. latifolia* (1%), and higher in *M. communis* and *Q. ilex,* (24% and 33%, respectively), compared to the spring values. *M. communis* had the lowest *g*_s_ decrease (64%) compared to the spring maximum, followed by *Q. ilex* (70%), *P. lentiscus* (75%), and *P. latifolia* (74%).

*R*_D_ was 43% and 12% higher than the spring maximum in *P. lentiscus* and *P. latifolia*, respectively, while *R*_D_ was 13% lower than the spring maximum in *Q. ilex*. There were no significant *R*_D_ differences in *M. communis* between spring and summer measurements. *P. lentiscus* and *Q. ilex* had the highest *R*_D_/*P*_N_ rate (0.94 ± 0.05 and 0.61 ± 0.06, respectively), followed by *P. latifolia* (0.56 ± 0.04), and *M. communis* (0.43 ± 0.02).

### Autumn measurements

*P*_N_ recovered 64% of the spring maximum in *Q. ilex*, *P. latifolia* and *M. communis* (mean value), and 53% in *P. lentiscus* at the end of September (*T*_max_ 28.2 ± 3.1°C) after the first rainfall (22.9 mm from the middle to the end of September) following drought. *g*_s_ recovered 59% of the spring maximum in *Q. ilex*, 54% in *M. communis,* 44% in *P. lentiscus,* and 35% in *P. latifolia.*

IWUE ranged from 100 ± 13 μmol (CO_2_) mol (H_2_O)^-1^ in *P. lentiscus* to 125 ± 13 μmol (CO_2_) mol (H_2_O)^-1^ in *P. latifolia. P. latifolia* had the highest *R*_D_ [1.7 ± 0.5 μmol (CO_2_) m^-2^ s^-1^], followed by *P. lentiscus* and *Q. ilex* [1.2 ± 0.3 μmol (CO_2_) m^-2^ s^-1^, mean value], and *M. communis* [0.6 ± 0.1 μmol (CO_2_) m^-2^ s^-1^]. In October, *P*_N_, *g*_s_ increased compared to the rates monitored in September in all the considered species while *R*_D_ increased in *P. latifolia*, *P. lentiscus* and *M. communis* and decreased in *Q. ilex.*

In September*, P. latifolia* had the highest *R*_D_/*P*_N_ ratio (0.17 ± 0.02) followed by *P. lentiscus* (0.15 ± 0.04), *Q. ilex* (0.12 ± 0.01) and *M. communis* (0.09 ± 0.02).

### Q_10_

*Q. ilex* and *P. latifolia* showed the highest Q_10_ value (1.78 ± 0.01, mean value) followed by *P. lentiscus* (1.47 ±0.03) and *M. communis* (1.44 ± 0.02).

### Leaf to air vapour pressure deficit

**Figure 3:**
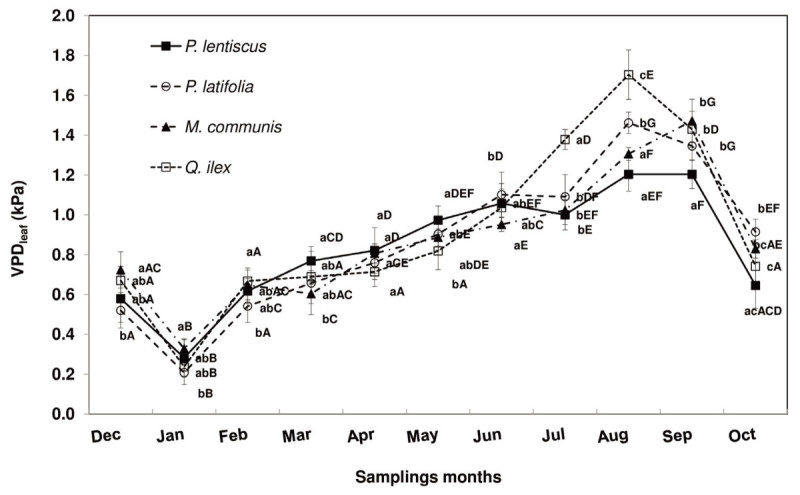
**Leaf to air vapor pressure deficit (VPD**_**leaf**_**) trend of** ***P. lentiscus*** **(** ***close squares*** **),** ***P. latifolia*** **(** ***open circles*** **),** ***M. communis*** **(** ***close triangles*** **) and** ***Q. ilex*** **(** ***open squares*** **) during the study period.** The mean values for each month (± SE) are shown (n = 40 leaves). Mean values with the same letters are not significantly different (p ≥ 0.05). Lowercase letters indicate the differences among the species for each month, capital letters indicate the intra-specific differences during the study period.

The seasonal VPD trend of the considered species is shown in Figure 3. The considered species had similar VPD trend with the lowest values in January ranging from 0.21 ± 0.06 kPa (in *P. latifolia*) to 0.33 ± 0.05 kPa (in *M. communis*). VPD increased from February (0.62 ± 0.06 kPa, mean value) to August (1.42 ± 0.21 kPa, mean value) when *Q. ilex* had the highest VPD (1.70 ± 0.12 kPa) and *P. lentiscus* the lowest one (1.20 ± 0.09). In September and October VPD, on an average, decreased by 7% and 45% respectively, compared to August.

### Statistical analysis

**Figure 4:**
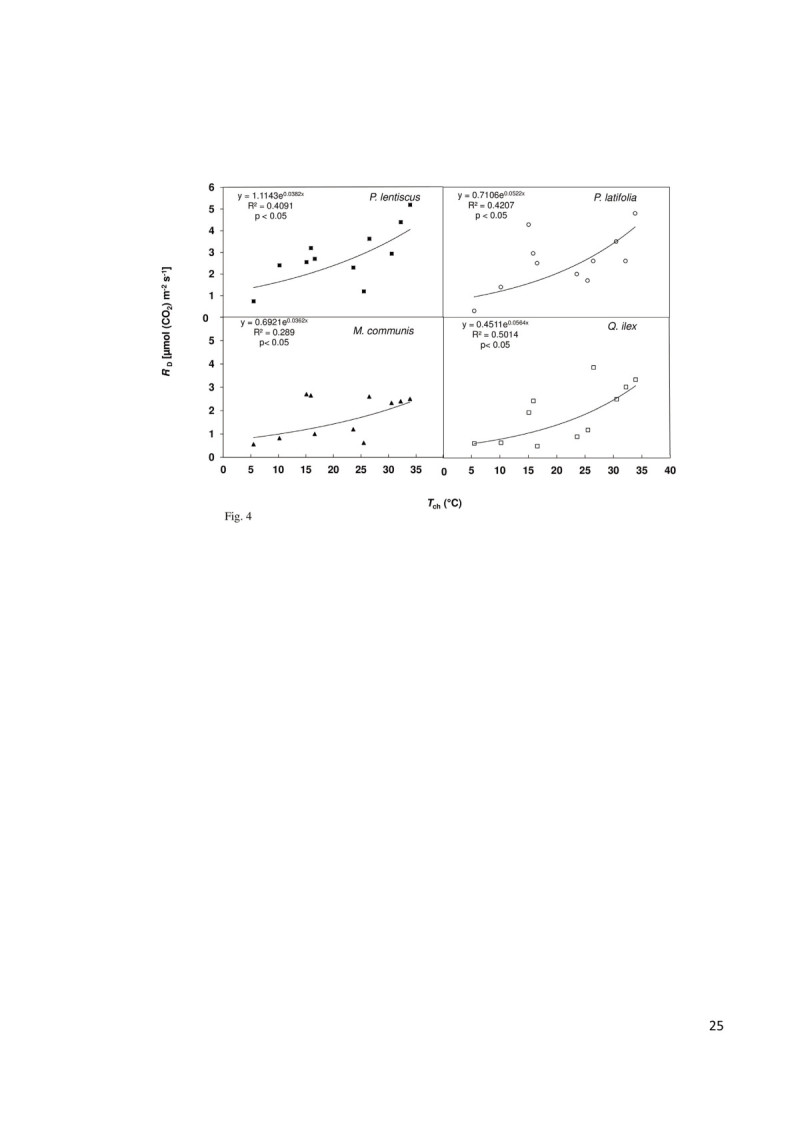
**Regression analysis between leaf respiration (** ***R***_**D**_**) and leaf chamber air temperature (** ***T***_**ch**_**) for the considered species.** Regression equation, determination’s coefficient (R^2^) and P-level are shown.

**Figure 5:**
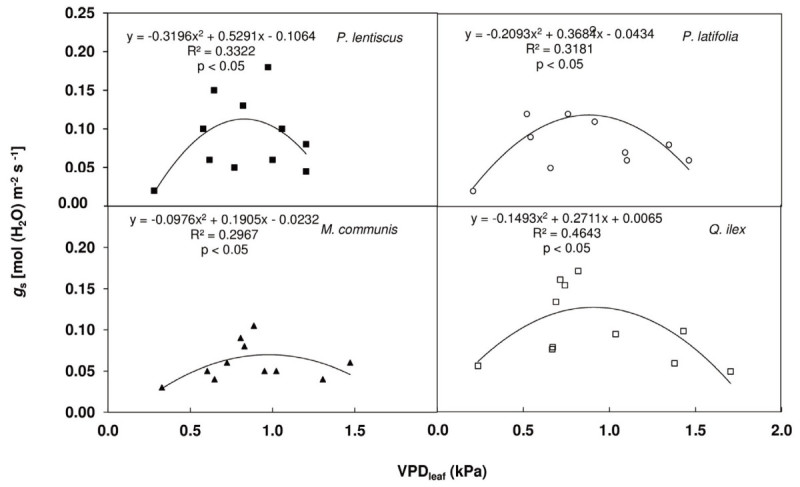
**Regression analysis between stomatal conductance (** ***g***_**s**_**) and leaf to air vapour pressure deficit (VPD**_**leaf**_**) for the considered species.** Regression equation, determination’s coefficient (R^2^) and P-level are shown.

The results of the regression analysis showed a significant relationship between *R*_D_ and *T*_ch_ and between *g*_s_ and VPD (Figures 4 and 5).

**Figure 6:**
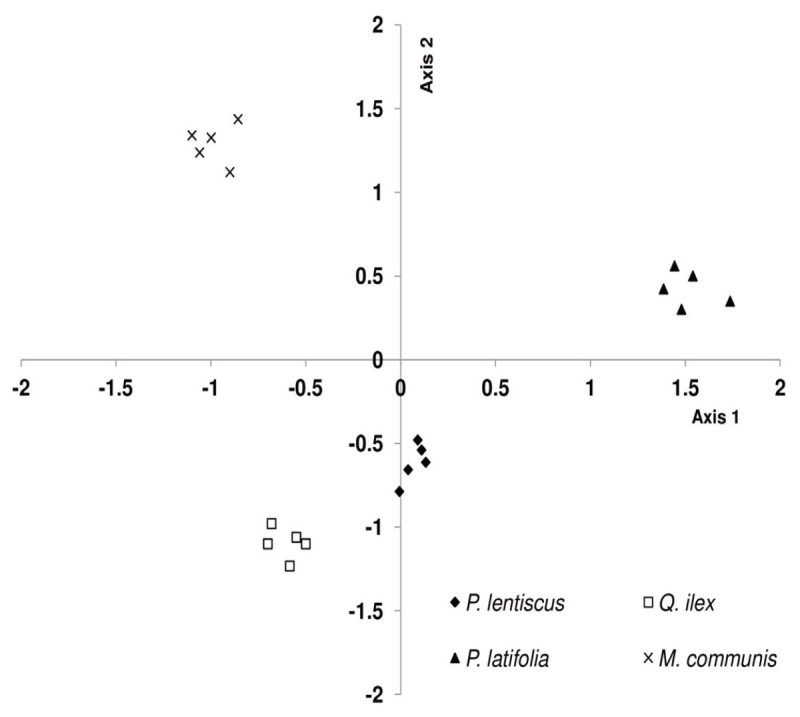
**Principal component analysis (PCA) carried out using physiological traits in winter, spring and summer (photosynthetic rates stomatal conductance transpiration rates intrinsic water use efficiency and leaf respiration) and morphological and anatomical leaf traits (LMA, LTD, leaf thickness, stomatal density, stomatal pore length and stomatal pore area) for the considered species.**

The PCA analysis extracted two factors accounting for 76% of the total variance among the considered species (48% and 28% for the 1^st^ and the 2^nd^ factor, respectively). The 1^st^ factor was related to physiological traits (*P*_N_ in summer, *g*_s_ in spring, IWUE in spring and summer, *R*_D_ in summer and spring) and anatomical leaf traits (L, SPL, SPA and SD). The 2^nd^ factor was mainly related to morphological leaf traits (LMA and LTD), and to *P*_N_ in winter and spring, and IWUE in winter. According to these results, the considered species were divided into three groups (Figure 6): the 1^st^ group included *P*. *lentiscus* and *Q. ilex*, the 2^nd^ group *P. latifolia*, and the 3^rd^ group *M. communis*.

## Discussion

Knowledge of plant species response to limited soil moisture is important for providing insights into potential ecological impacts on wild populations (Wu et al. 2010) also in consideration of climate change scenarios which hypothesizes an increasing aridity in many regions worldwide (Canadell et al. 2007). Our results on the whole underline different morphological, anatomical and physiological leaf traits of the considered shrub species which are indicative of their adaptive capability to Mediterranean climate stress factors. Among the considered species, *Q. ilex* has the highest *P*_N_ in spring associated to the highest *R* which may be related to the concomitance of vegetative activity (spring shoots production) and flowering (Gratani et al. 1996). *Q. ilex* has the largest tolerance to low winter air temperatures evidenced by the lowest *P*_N_ decrease (68% of the maximum) and low *R*_D_ rates (87% of the maximum). Under drought conditions, plants optimize carbon assimilation and minimize water loss by decreasing *g*_s_ (Medrano et al. 2002), and IWUE may be considered a good indicator of carbon assimilation optimization. *Q. ilex* has a high responsiveness to drought showing a high *g*_s_ decrease at the beginning of June (41% lower compared to the maximum) associated with a 22% *P*_N_ decrease determining a 40% IWUE increase compared to the maximum. The responsive stomatal behaviour is also underlined by the significant relationship between *g*_s_ and VPD (R^2^ = 0.46). As drought stress progresses in July, *Q. ilex* IWUE does not significantly increase because of *P*_N_ and *g*_s_ change to the same extent. At the highest drought intensity (August) IWUE decreases by 10% compared to June, due to a higher *P*_N_ decrease than *g*_s_. Despite the high *P*_N_ decrease in August (by 65% compared to the maximum), *Q. ilex* is able to recover 65% of the spring rates in September. Gratani and Varone (2003) underline the sufficiently high leaf water potential and relative water content during drought in *Q. ilex*. Moreover, the results underline that *Q. ilex* does not seem to suffer significant metabolic damage that could make a demand on respiratory products as drought stress progresses, according to the results of Rodríguez-Calcerrada et al. (2011). This is also pointed out by a 13% *R*_D_ decrease in August compared to the spring rates. Due to the high *P*_N_ decrease, *Q. ilex* shows a relatively high *R*_D_/*P*_N_ ratio (0.61 ± 0.06) in August. The most important factor determining how negative the plant carbon balance becomes under water stress is the absolute and proportional change in *P*_N_ rates since drought has typically a greater proportional inhibitory effect on photosynthesis than on respiration thus, resulting in a higher *R*_D_/*P*_N_ ratio (Galmés et al. 2007). As regards leaf anatomy and morphology, *Q. ilex* high SD and low SPL and SPA, associated to a high LMA and LTD, contribute to an efficient control of gas exchange. Niinemets (2001) underlines that the adaptive significance of leaves characterised by thick cell walls and low fractions of intercellular air spaces (i.e. high LMA and LTD) lies in their large elastic module which upholds water flow from drying soils.

*P. lentiscus* strategy to stress factors is similar to that of *Q. ilex* (i.e. high *P*_N_ in spring and a relatively high *P*_N_ in winter associated to a high *R*_D_). In August *g*_s_ and *P*_N_ decrease by 72% and 63%, respectively, and *R*_D_ increases by 43% resulting in a high *R*_D_/*P*_N_ ratio (0.94 ± 0.05).

The similar strategy of *P. lentiscus* and *Q. ilex* is also underlined by their similar IWUE values during the study period and their *P*_N_ recovery capability in September. Moreover, *P. lentiscus* shows a higher relationship between *g*_s_ and VPD (R^2^ = 0.33) as well as *Q. ilex*. A higher *P*_N_ recovery capability might be related to the capacity of this species to have low leaf water potential and relative water content variations during the year (Gratani and Varone 2004). At morphological and anatomical levels, *P. lentiscus* is characterised by a high LMA and LTD. In particular, the larger SPL, SPW and SPA in *P. lentiscus* with respect to *Q. ilex* may be related to its origin from the semi-arid steppes of central Asia with an exceptionally hot summer and an exceptionally cold and dry winter (Blondel and Aronson 1999). Billing et al. (1971) and Cunningham and Read (2003) hypothesize that plant species which have originated in climates with more fluctuating temperatures may have a higher gas-exchange acclimation to air temperature than those originated in more constant climate. Thus, *Q. ilex* and *P. lentiscus* capability to maintain sufficiently high photosynthetic rates both in cold and drought stress periods seem to be related to their origin under a climate characterized by a pronounced seasonality.

Compared to the considered species, *P. latifolia* has the lowest *P*_N_ decrease in drought (45% compared to the spring maximum) associated to a 74% *g*_s_ decrease resulting in a high IWUE. The lower *R*_D_/*P*_N_ ratio (0.56 ± 0.04) in *P. latifolia* compared to *P. lentiscus* and *Q. ilex*, is due to the lowest *P*_N_ decrease in drought. The high *P. latifolia* photosynthetic recovery capacity in September (64% of the maximum) after the first rainfall following drought attests to its greater drought tolerance through the maintenance of a high *P*_N_ rate even at low leaf water potential (Bombelli and Gratani 2003). Moreover, the high *P. latifolia* LMA, due to the presence of thick cell walls and sclereids (Gratani and Bombelli 1999) and the high LTD (i.e. a densely packed mesophyll cells with few air spaces, Gratani and Bombelli 2000) contribute to improve drought resistance by improving water use efficiency (Niinemets 2001) and limiting photochemical damage to the photosynthetic apparatus through the reduction of the incident irradiance (Jordan et al. 2005). On the contrary, the lower *P. latifolia P*_N_ in winter compared to the maximum underlines its lower tolerance to cold temperatures, according to the results of Ogaya and Peñuelas (2003), and Ogaya et al. (2011), also pointed out by the highest *R*_D_/*P*_N_ (0.75 ± 0.03). The lowest *R*_D_ rates in January underline the limitation of the enzyme activity of the respiratory apparatus (i.e. glycolysis, the TCA cycle and mitochondrial electron transport chain) (Atkin and Tjoelker 2003).

*M. communis* has a physiological response to drought similar to that of *P. latifolia*, which may be related to their common origin in the dry tropics of the continental Africa and adjacent regions (Blondel and Aronson 1999). *M. communis* has a low *P*_N_ decrease (by 46%) during drought associated to stable *R*_D_ rates which determine a lower *R*_D_/*P*_N_ ratio (0.43 ± 0.02). In winter a 92% *P*_N_ decrease associated to a 79% *R*_D_ decrease results in a higher *R*_D_/*P*_N_ ratio (0.61 ± 0.02). Hernández et al. (2010) underline that *M. communis* has a low capacity to transport water from roots to leaves also under water availability. Gratani et al. (1980) show its low biomass production capability respect to other Mediterranean shrubs which are pointed out by the significant lowest *M. communis P*_N_ rates during the study period compared to the other considered species. Moreover, the low stomatal control of *M. communis* is pointed out by a lower relationship between *g*_s_ and VPD (R^2^ = 0.29). Despite the highest SD, *M. communis* has a very small SPL and SPW which could explain the low *g*_s_. The lower LMA and LTD *M. communis* with respect to *P. latifolia* underline a lower leaf consistency. The above considerations are confirmed by the PCA showing a higher similarity between *Q. ilex* and *P. lentiscus* compared to *P. latifolia* and *M. communis*.

Chu et al. (2011) suggest that *R*_D_/*P*_N_ ratio can be considered as a simple approach to leaf carbon balance because it indicates the percentage of photosynthates that is respired. Our results show that *R*_D_/*P*_N_ ratio of the considered species, calculated over the study period, varies from 0.15 ± 0.04 in autumn, 0.24 ± 0.05 in spring, through 0.29 ± 0.15 in winter to 0.46 ± 0.11 in summer, and it is indicative of the different sensitivity of both *R*_D_ and *P*_N_ to water availability and air temperature changes, according to results of Zaragoza-Castells et al. (2008). The low *R*_D_/*P*_N_ ratio in autumn and spring of the considered Mediterranean evergreen species (i.e. during vegetative activity) underlines the highest *P*_N_ rates during the favorable periods, when resources are not limited, and leaves take in roughly three to five times more CO_2_ than they lose by dissimilatory processes during the same period of time (Larcher 2003). On the contrary, the highest *R*_D_/*P*_N_ ratio in summer underlines the lower sensitivity of respiration to drought (Atkin and Macherel 2009) that is indicative of a higher proportion of fixed carbon which is respired at elevated temperature (Gratani et al. 2011; Riikonen et al. 2012). Thus, summer drought can reduce the carbon assimilation because of *R*_D_ rates increasing more than *P*_N_ rates.

It is known that over short-term rises in temperature, *R*_D_ increases exponentially but the seasonal temperature sensitivity of *R*_D_ is often lower than that observed over hours, a phenomenon known as thermal acclimation (Rodríguez-Calcerrada et al. 2012). This phenomenon involves adjustments in *R*_D_ rates to compensate for changes in air temperature (Atkin et al. 2000). In particular, acclimation of *R*_D_ to high temperatures can result in a lower slope (i.e. lower Q_10_) for the temperature-response curve of acclimated tissue (Atkin et al. 2000). Among the considered species, *M. communis* has the higher acclimation to high temperatures compared to the other species pointed out by the lower Q_10_ value (1.44 ± 0.02) and by more stable *R*_D_ rates during the year. There is growing evidence that acclimation of *R*_D_ to heat and drought reflects the metabolic down-regulation that reduces carbon depletion and helps plants to grow and survive in Mediterranean-type environments (Rodríguez-Calcerrada et al. 2010, 2011). Understanding the function of plant species in water limited environments is crucial in order to make informed land management decisions (Maseyk et al. 2008). Moreover, under a Mediterranean type of climate, our results underline the importance of including seasonal variations of photosynthesis and respiration in carbon balance models.

## Conclusions

Limitations to plant growth imposed by the Mediterranean climate are mainly due to carbon balance in response to stress factors. In particular, water stress associated to high air temperature and irradiance in summer causes a marked decrease in CO_2_ assimilation. The results underline the response of the evergreen species co-occurring in the Mediterranean maquis to Mediterranean stress factors. In particular, the lower *R*_D_/*P*_N_ in autumn and spring underlines the highest *P*_N_ rates during the favorable periods while the highest *R*_D_/*P*_N_ ratio in summer shows the lower sensitivity of respiration to drought. Among the considered species, *Q. ilex* and *P. lentiscus* have the largest tolerance to low winter temperatures while *P. latifolia* and *M. communis* to drought. Among the considered species, *M. communis* has the higher acclimation to high temperatures compared to the other species and this is underlined by the lower Q_10_ value and the more stable *R*_D_ rates during the year. The predicted global warming might differently affect carbon balance of the considered species, with a possible change in Mediterranean shrublands composition in the long term.

## Authors’ original submitted files for images

Below are the links to the authors’ original submitted files for images. Authors’ original file for figure 1 Authors’ original file for figure 2 Authors’ original file for figure 3 Authors’ original file for figure 4 Authors’ original file for figure 5 Authors’ original file for figure 6

## Abbreviations

DM: Leaf dry mass
gs: Stomatal conductance
L: Leaf thickness
LA: Leaf surface area
LMA: Leaf mass area
LTD: Leaf tissue density
SD: Stomatal density
SPA: Stomatal pore area
SPL: Stomatal pore length
SPW: Stomatal pore width
PN: Net photosynthetic rate
RD: Respiration rate
Tch: Leaf chamber air temperature
Tl: Leaf temperature
IWUE: Intrinsic water use efficiency.
