# Supplementary figures and images for: Morphological, anatomical and physiological leaf traits of Q. ilex, P. latifolia, P. lentiscus, and M. communis and their response to Mediterranean climate stress factors
Source: Bot Stud. 2013 Sep 17;54:35. doi: 10.1186/1999-3110-54-35 (PMC5432955; doi:10.1186/1999-3110-54-35)

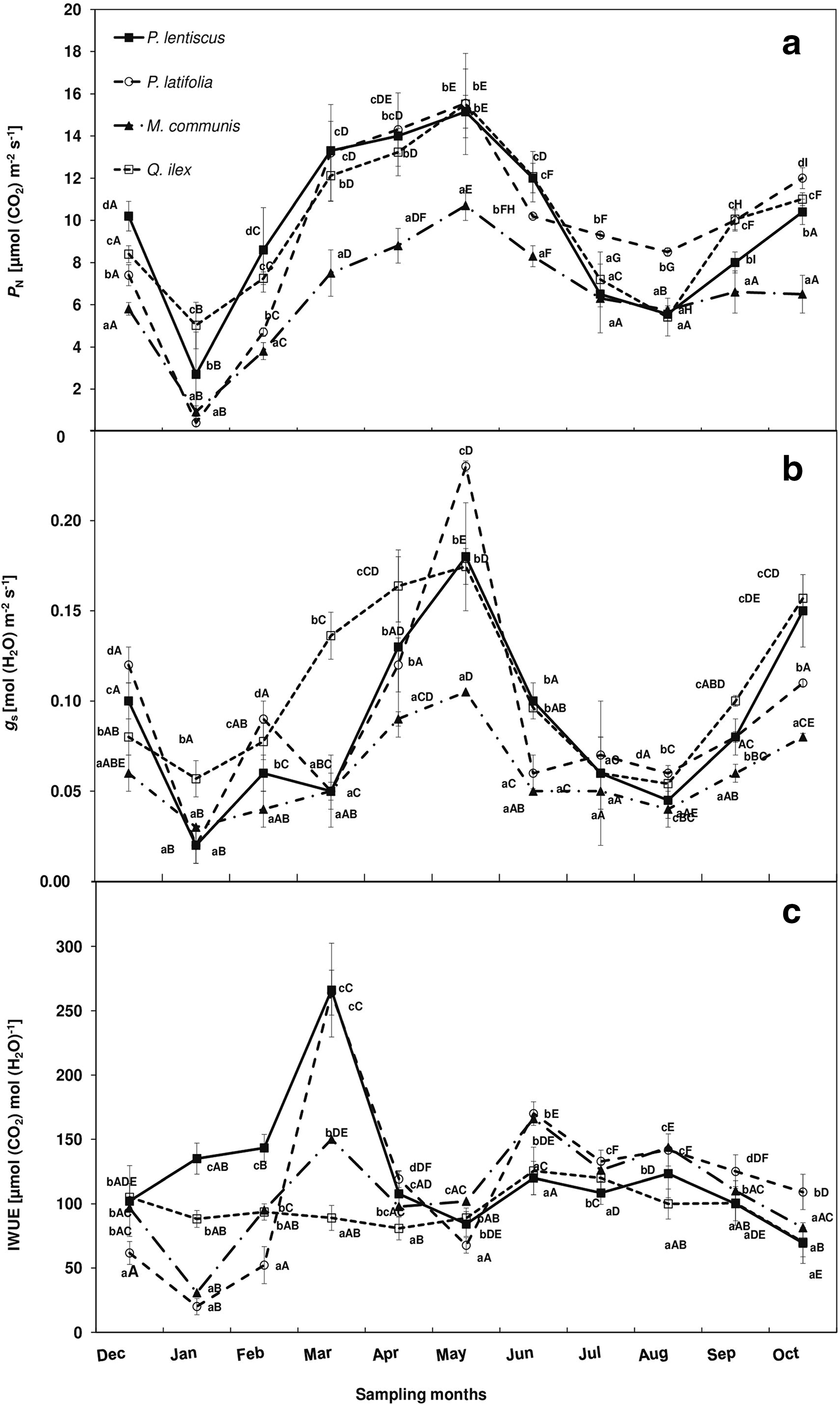

Supplement: Supplementary file 1 — Authors’ original file for figure 1 [file 40529_2011_32_MOESM1_ESM.tif]

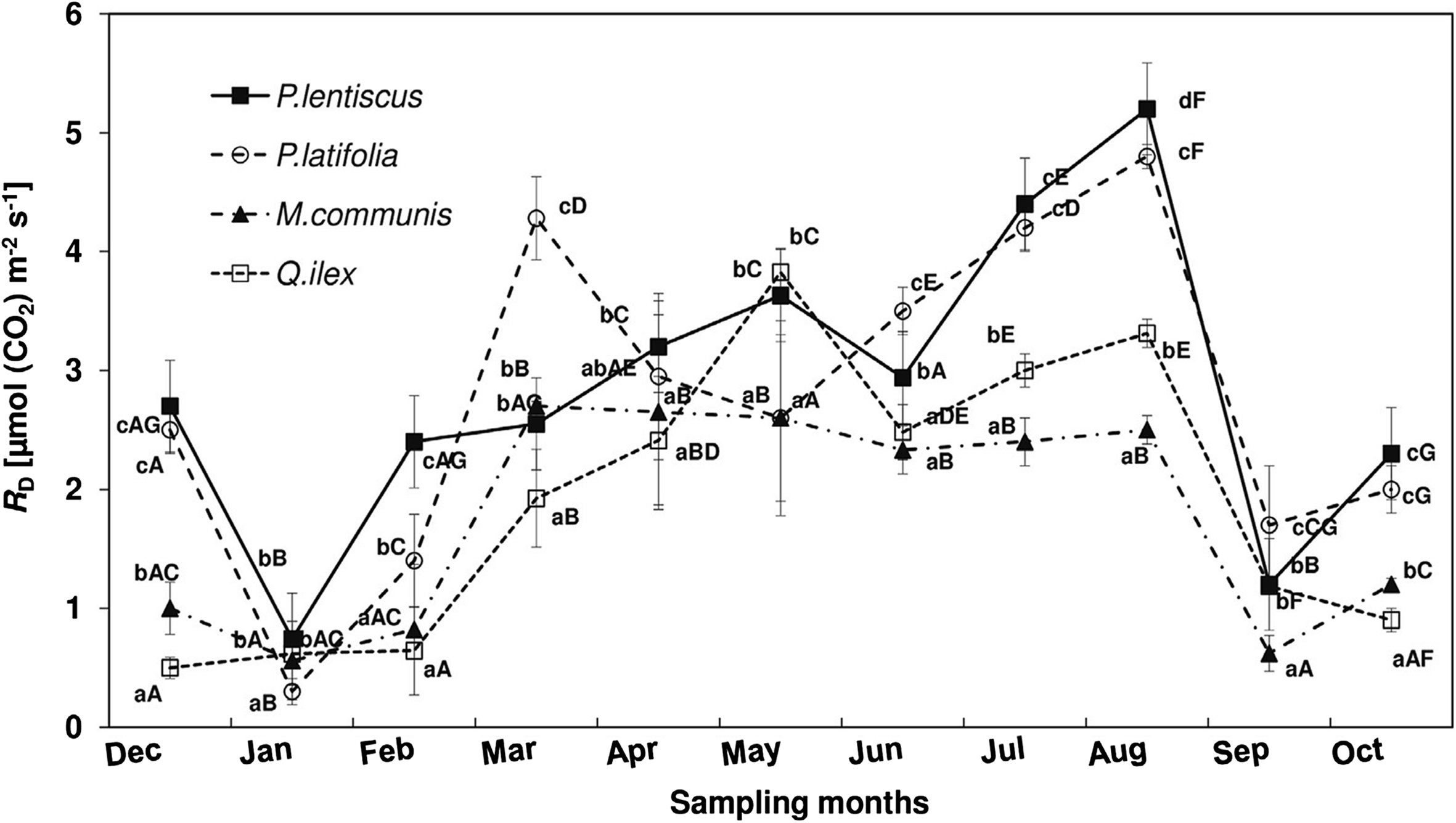

Supplement: Supplementary file 2 — Authors’ original file for figure 2 [file 40529_2011_32_MOESM2_ESM.tif]

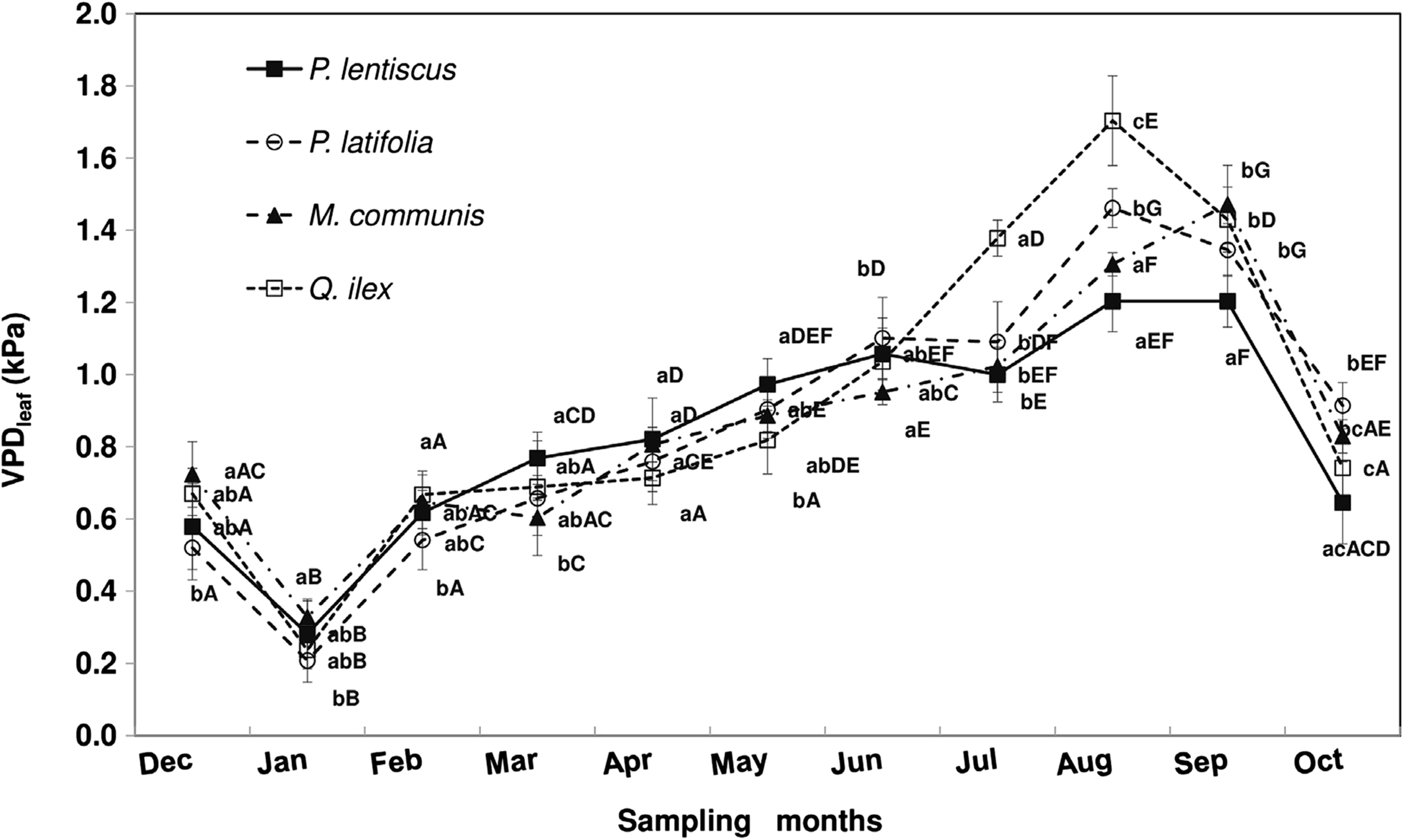

Supplement: Supplementary file 3 — Authors’ original file for figure 3 [file 40529_2011_32_MOESM3_ESM.tiff]

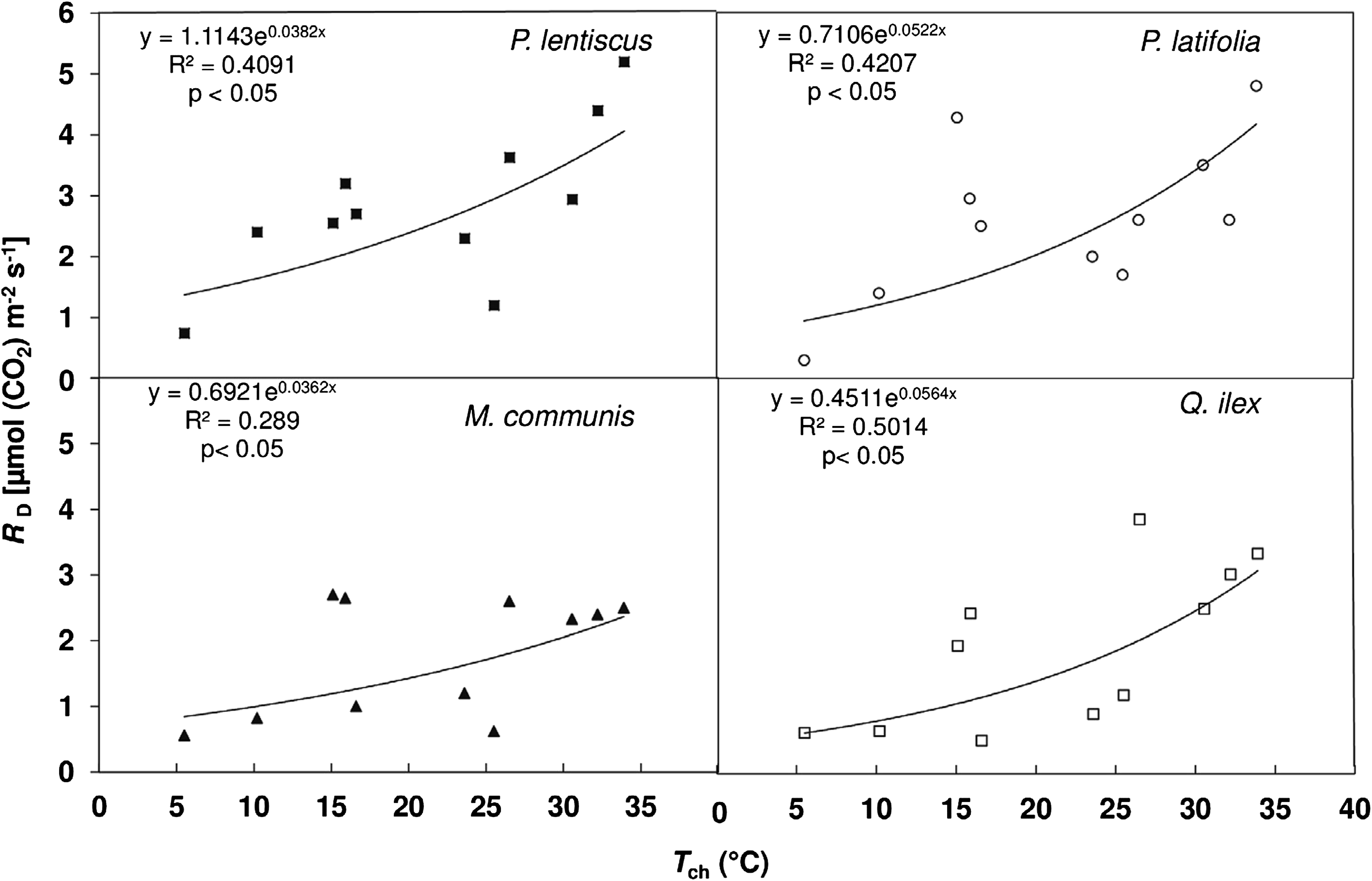

Supplement: Supplementary file 4 — Authors’ original file for figure 4 [file 40529_2011_32_MOESM4_ESM.tif]

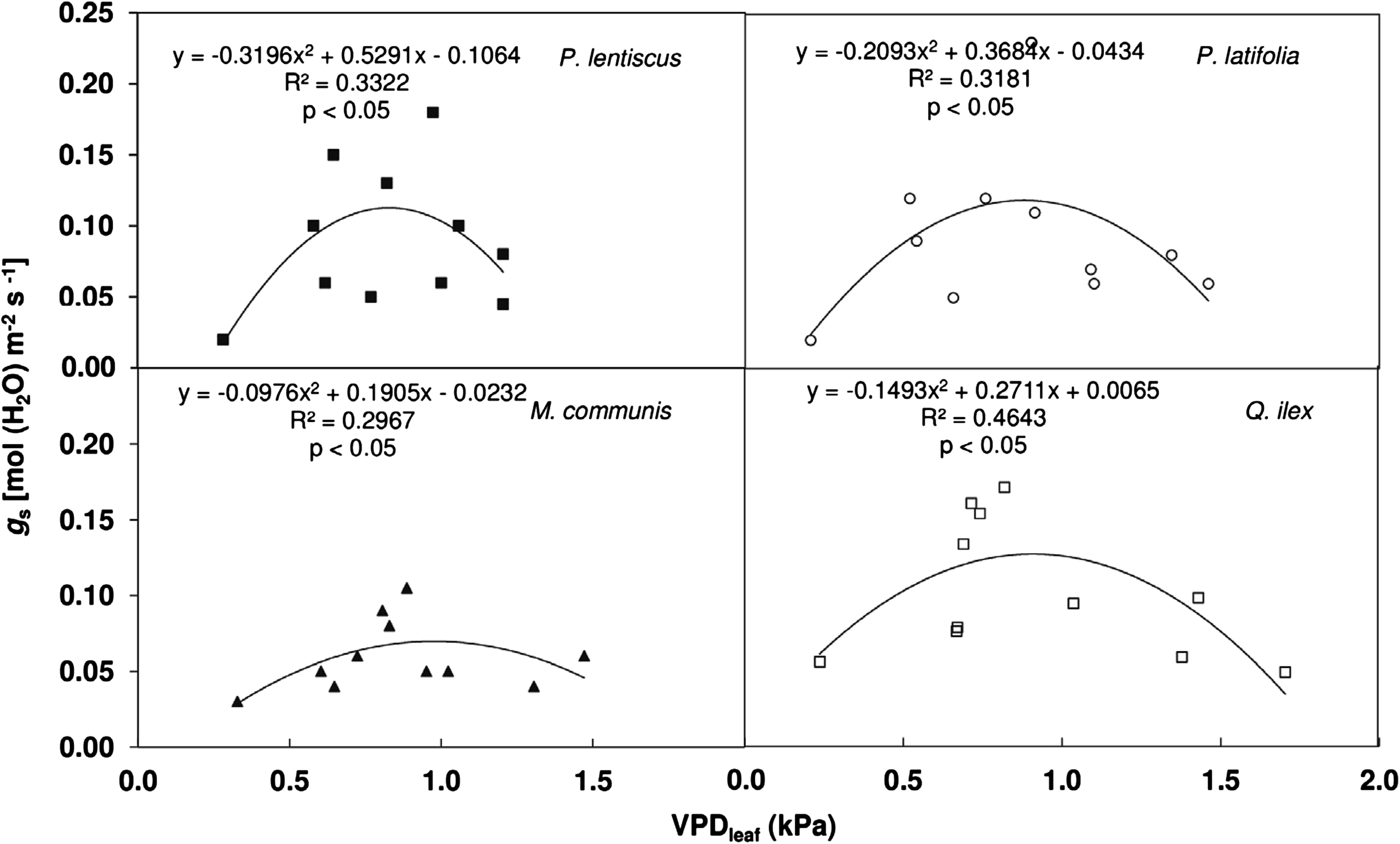

Supplement: Supplementary file 5 — Authors’ original file for figure 5 [file 40529_2011_32_MOESM5_ESM.tif]

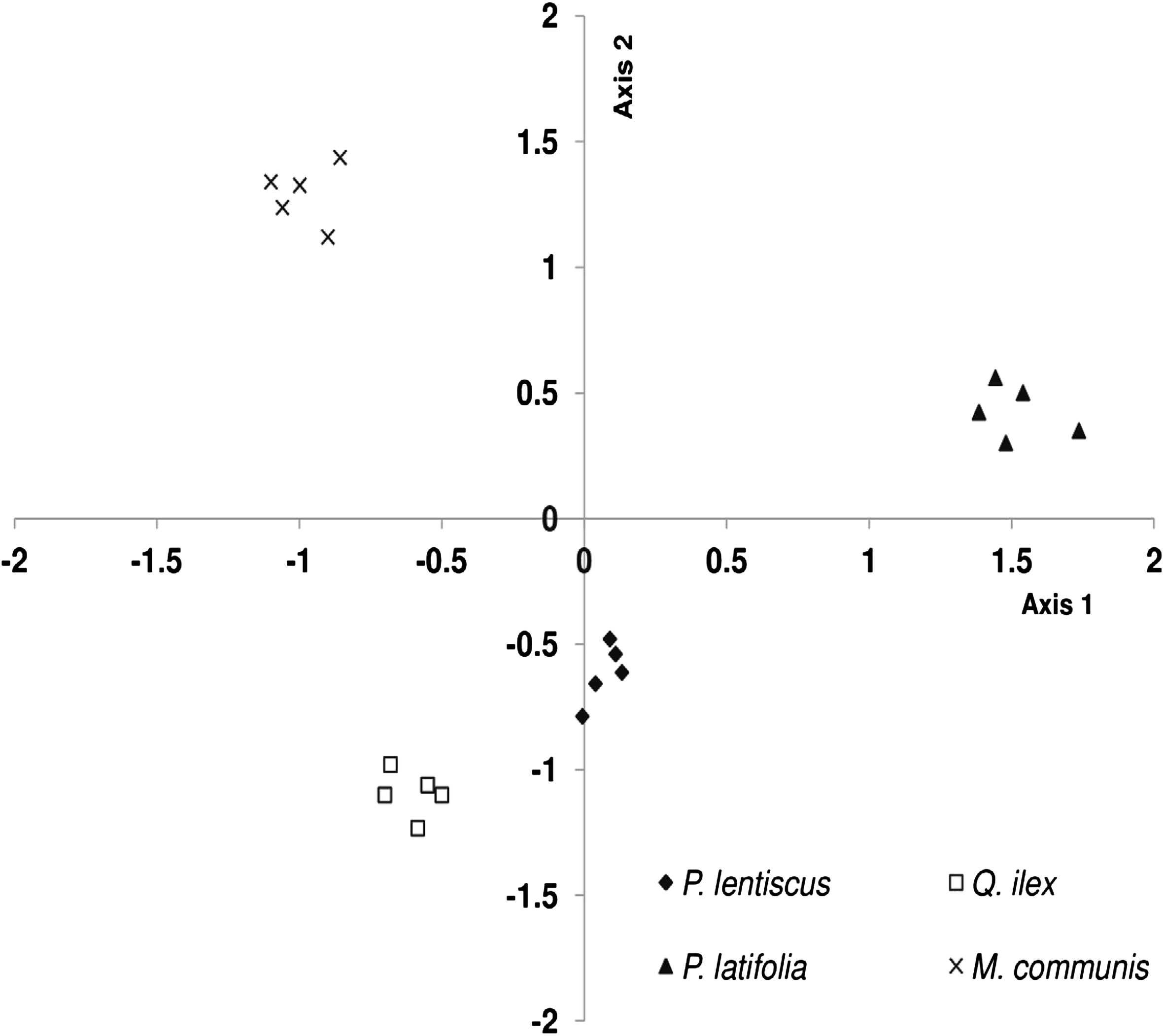

Supplement: Supplementary file 6 — Authors’ original file for figure 6 [file 40529_2011_32_MOESM6_ESM.tif]
